# Detection and phylogenetic analysis of blood-associated pathogens from spleen samples of wild raccoons (*Procyon lotor*) in Germany

**DOI:** 10.1038/s41598-024-82581-7

**Published:** 2024-12-28

**Authors:** Maria Sophia Unterköfler, Aria Schwingshandl, Barbara Eigner, Jutta Pikalo, Josef Harl, Joachim Spergser, Peter Steinbach, Diana Jeschke, Michael Striese, Elisabeth Striese, Hermann Ansorge, Hans-Peter Fuehrer, Mike Heddergott

**Affiliations:** 1 Department of Biological Sciences and Pathobiology, Institute of Parasitology, University of Veterinary Medicine Vienna, Vienna, Austria; 2 Department of Biological Sciences and Pathobiology, Institute of Pathology, University of Veterinary Medicine Vienna, Vienna, Austria; 3 Department of Biological Sciences and Pathobiology, Institute of Microbiology, University of Veterinary Medicine Vienna, Vienna, Austria; 4 Faculty of Chemistry, University of Göttingen, Göttingen, Germany; 5 Department of Zoology, Musée National d’Histoire Naturelle, Luxembourg, Luxembourg; 6 Senckenberg Museum of Natural History Görlitz, Görlitz, Germany; 7 International Institute Zittau, Technical University Dresden, Zittau, Germany

**Keywords:** Vector-borne disease, Invasive raccoon, Haemotrophic *Mycoplasma*, Parasitology, Pathogens

## Abstract

Raccoons (*Procyon lotor*) originated in North America and have been introduced to Europe. Due to their close contact with human settlements, they are important reservoirs for zoonotic pathogens, such as *Baylisascaris procyonis*. The relevance and prevalence of vector-borne pathogens have not yet been fully elucidated. In this study, we screened 285 spleen samples of raccoons, collected between 2019 and 2022 in Germany. The samples were analysed by PCR to detect *Mycoplasma* spp., Anaplasmataceae, *Bartonella* spp.*, Babesia* spp., *Rickettsia* spp., Filarioidea, Trypanosomatida and *Hepatozoon* spp., and positive PCR products were sequenced. In total, 104 samples were positive for *Mycoplasma* spp. (36.49%), making this the first study to detect *Mycoplasma* spp. in raccoons outside of North America. Three samples were positive for *Babesia* spp. (1.05%) and two for *Anaplasma phagocytophilum* (0.7%). Phylogenetic analysis revealed that the *Mycoplasma* spp. detected all belong to the haemotrophic mycoplasmas cluster and were grouped within a single phylogenetic clade. Two different *Babesia* spp. were detected, one of which was closely related to *Babesia canis*, while the other was more closely related to *Babesia* sp. from ruminants. It is unclear whether the pathogens detected have an impact on the health of raccoons or whether they may serve as a reservoir for other animals.

## Introduction

Raccoons (*Procyon lotor*) are medium-sized carnivores with an original range in Northern and Central America^1^. They have been accidentally or deliberately introduced into several Asian and European countries since the 1920s^2^. Within Europe, they are mainly distributed in Central Europe, particularly Germany and the neighbouring countries, but there are also populations in Spain, Italy, Belarus, and Russia^2,3^. Recent genetic studies have shown that the populations can be traced back to several separate introduction events^4,5^. In the last few decades, population density and geographical distribution of the raccoon have greatly increased in Germany, including a spread into urban areas, where they live in close proximity to humans and pets. They could thus represent important reservoirs for pathogens relevant to humans and their animals^6,7^. For example, the zoonotic nematode *Baylisascaris procyonis* can cause severe neurological disease in humans^8^ and the canine distemper virus can infect dogs, causing severe disease^9^. In addition, as medium sized carnivores, they could have an impact on the ecosystem by decimating prey or by competing for resources with native carnivores. However, this has been poorly studied in Europe and robust data is not available to support or reject this hypothesis^2,7^.

Blood-associated pathogens are being studied increasingly in raccoons. Haemotrophic mycoplasmas, also known as haemoplasmas, have been detected in raccoons from North America and crab-eating raccoons (*Procyon cancrivorus*) from South America^10,11^. This highly specialized and unique group of bacteria within the genus *Mycoplasma* target erythrocytes and can cause acute or chronic anaemia in several mammalian species, including humans^12^. The bacterium *Anaplasma phagocytophilum* is transmitted by ticks and can infect various mammals, including humans^13^. In raccoons, it has been detected with varying prevalence^14–18^. Other blood-associated bacteria detected in raccoons include *Ehrlichia* spp., *Bartonella* spp., and *Rickettsia* spp.^14,19–22^.

*Babesia* spp. are transmitted by ticks and can cause haemolysis in their mammalian hosts. Most species are considered specific to their hosts, but *Babesia microti*, for example, is known to infect different species and can also infect humans^23^. Five different *Babesia* genotypes have been found in raccoons, one of which has so far only been detected in Japan, where raccoons are not native^24,25^. Other unicellular blood parasites found in raccoons in Europe and the Americas are *Hepatozoon* spp. and *Trypanosoma cruzi*^14,19,26–28^.

In Europe, *Dirofilaria* spp. are known to be endemic in Southern Europe and are extending their distribution ranges northwards^29^. They are transmitted by mosquitoes and mainly affect dogs (*Canis lupus familiaris*), but also cats (*Felis catus*), humans, and a variety of wild mammals, such as foxes (*Vulpes vulpes*), jackals (*Canis aureus*) and wolves (*Canis lupus*). In dogs, adult *D. repens* form subcutaneous nodules, while adult *D. immitis* are found in the cardiopulmonary arteries and have, therefore, a higher impact on the animals’ health^30^. *Dirofilaria* spp. so far have not been detected in raccoons from Europe^31,32^. However, Northern American raccoons and Southern American crab-eating raccoons are hosts for *D. tenuis* and *D. cancrivori*, and even a case of *D. immitis* infection has been reported^33–35^.

The role of raccoons as reservoirs for blood-associated pathogens has been investigated only recently. For most pathogens, it is unclear which species occur in raccoons, how prevalent they are and what impact they can have on other wild or domestic animals or humans. This study aims to determine the species of blood-associated pathogens and their prevalence in German populations of raccoons.

## Results

### Prevalence

**Fig. 1:**
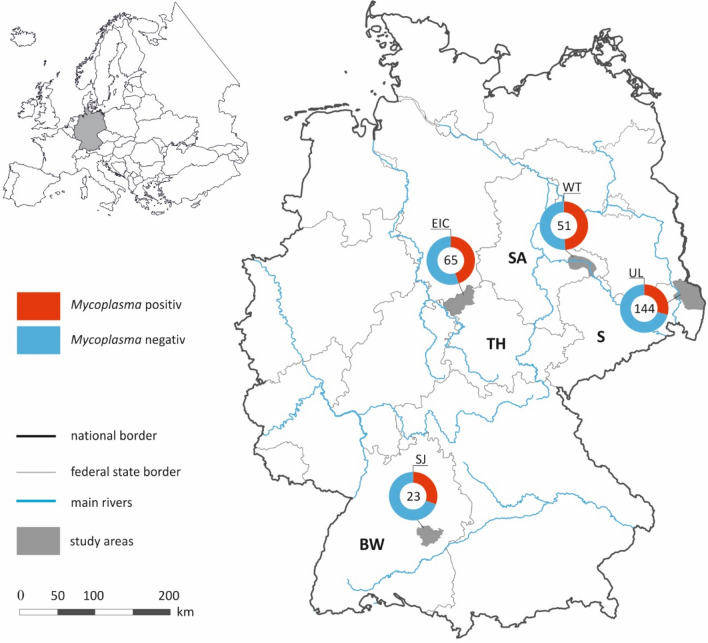
Geographical location and number of raccoons (*Procyon lotor*) sampled in Germany and the prevalence of *Mycoplasma* spp. EIC, Eichsfeld; TH, Thuringia, WT, Wittenberg, SA, Saxony-Anhalt, UL, Upper Lusatia, S: Saxony, SJ: Swabian Jura, BW: Baden-Württemberg.

Of the 285 individuals tested, 105 (36.84%) were positive for at least one pathogen. Of these, 52 were male (i.e. 34.21% of males positive), 51 were female (i.e. 38.64% of females positive), and two were of unknown sex. The most prevalent pathogens were *Mycoplasma* spp. with 104 (36.49%) positive samples. Prevalence was 49.02% in Saxony-Anhalt, 44.62% in Thuringia, 30.43% in Baden-Württemberg and 29.16% in Saxony (Fig. 1). Three samples (1.05%) collected in Saxony were positive for *Babesia* spp., and two (0.7%) samples collected in Saxony and Thuringia were positive for *Anaplasma phagocytophilum*. Co-infections were detected with *Babesia* spp. and *Mycoplasma* spp. (n = 2), and *A. phagocytophilum* and *Mycoplasma* spp. (n = 1). Filarioidea, Trypanosomatida, *Bartonella* spp., and *Rickettsia* spp. were not detected.

### Phylogenetic analysis

**Fig. 2:**
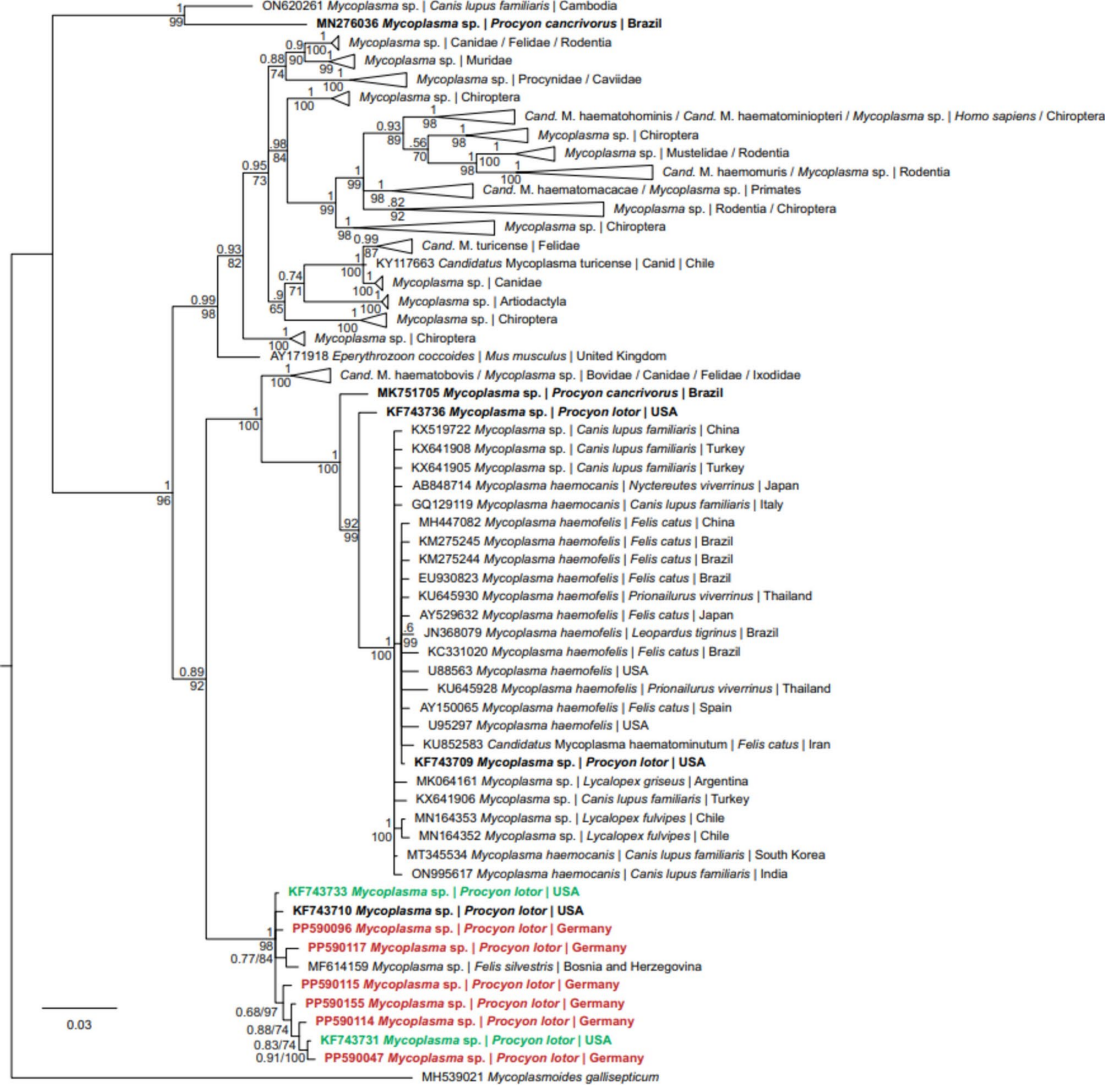
Bayesian interference tree featuring *16S rRNA* (538 nucleotide positions) sequences of selected *Mycoplasma* spp. Nodes are marked with Bayesian interference posterior probabilities and maximum likelihood bootstrap values. Sequences which are written in bold are from *Procyon* spp., sequences marked in red were obtained in this study, and sequences marked in green are identical to sequences obtained in this study. Triangles represent collapsed clades. The scale bar indicates the expected mean number of substitutions per site according to the model of sequence evolution applied.

Phylogenetic analysis was performed for *Mycoplasma* spp. and *Babesia* spp. because species identification was not possible by comparison with published sequences. All sequences used for the phylogenetic analysis were uploaded to NCBI GenBank under the accession numbers PP590044- PP590159 (*Mycoplasma* spp.) and PP577796- PP577798 (*Babesia* spp.). Three *Mycoplasma* spp. and the two *A. phagocytophilum* sequences were of low quality and therefore not uploaded. The quality of the remaining sequences was high. However, peak overlays were detected in 15 sequences at the position where haplotypes differed from each other. This was interpreted as mixed haplotype infection of the same *Mycoplasma* species. By comparing with the remaining sequences, the haplotypes could be determined, and separated sequences were uploaded for these samples. In the phylogenetic tree, all *Mycoplasma* spp. sequences were placed within the haemotrophic cluster of genus *Mycoplasma* and grouped together within the same clade (Fig. 2). Two phylogenetically distinct *Babesia* spp. sequences were detected (Suppl. 1). One sequence (n = 1) was closely related to *Babesia canis* sequences while the other sequence (n = 2) was more closely related to *Babesia* spp. from ruminants.

## Discussion

In the present study, we detected a high prevalence of haemotrophic mycoplasmas in raccoons from Germany. A high prevalence of these specialized mycoplasmas has also been reported in other wild animals, but captive animals usually are less likely to be infected^36,37^. While haemotrophic mycoplasmas can cause severe disease in humans and domestic animals, the impact on the health of wild animals is not clear^12,36,38,39^. Wild animals are most likely asymptomatic carriers of these host-specific haemotrophic mycoplasmas, although cross-infection of related hosts and zoonotic infections of humans cannot be excluded^36,38^.

In a worldwide meta-analysis, no regional differences were observed regarding the prevalence of haemotrophic mycoplasmas in wild animals^36^. However, in the present study, performed in Germany, we found regional differences between the four sampling regions, with highest prevalence in Saxony-Anhalt (49.02%) and lowest prevalence in Saxony (29.16%). These differences may only be detectable on this small geographic scale. In addition, the prevalence of haemotrophic mycoplasmas seems to be higher in undisturbed environments, and the different bacterial loads might, therefore, reflect the impact of humans on the habitats^36^. In the present study this was also seen when comparing the proportion of settlement and transport area to total area, which was 5.4–11% in Wittenberg, Saxony-Anhalt and Eichsfeld, Thuringia, these being the regions with a higher *Mycoplasma* prevalence (49.02% and 44.62% respectively) compared to 11.1–17.1% in Swabian Jura, Baden-Württemberg and Upper Lusatia, Saxony (30.43% and 29.16% respectively)^40^.

This is the first study in Europe to detect haemotrophic mycoplasmas in raccoons, and there has only been one study in North America to date that reported a prevalence of 62.1%, which was higher than in our study at a prevalence of 36.5%^10^. The number of clades within the haemotrophic mycoplasmas cluster was also lower, as we could only identify one clade, clustering with one of the haemotrophic *Mycoplasma* sequences reported from Georgia, USA. It is thus possible that only one haemotrophic *Mycoplasma* species was introduced into Europe along with the raccoons^10^. However, several lineages within this clade were detected in our study. This indicates that this haemotrophic *Mycoplasma* species was either introduced several times or that it was already present in Europe before the introduction of raccoons, as it was also previously detected in a European wildcat (*Felis s. silvestris*) in a raccoon-free area^41^.

The haemotrophic *Mycoplasma* found in raccoons in Germany were phylogenetically closely related to a haemotrophic *Mycoplasma* detected in a wildcat from Bosnia and Herzegovina^41^. However, it is not clear whether this *Mycoplasma* sp. can cross species or can cause symptoms in wildcats. Alternatively, the pathogen could have been present in the blood only transiently, which would argue against an actual infection, similar to the previous detection of *M. ovis* in a wildcat^37^.

In contrast to the prevalence of *Mycoplasma* spp. (36.49%), the prevalence of *Babesia* spp. (1.05%) and *A. phagocytophilum* (0.7%) was low, and co-infections were only detected in three animals. Reasons for the low pathogen prevalences in raccoons could be either that the raccoon is not a particularly suitable host, the arthropod vector prefers other animals, or the overall occurrence of these parasites in this region is low. While cervids show a high rate of infection with *A. phagocytophilum* (> 90%), dogs in Germany rarely harbour *B. canis* (0.7%)^42–44^. In future studies, the occurrence of *B. canis* in raccoons should be investigated in regions where it is known to be more prevalent in order to evaluate its role as a reservoir host. In the present study, we detected a *Babesia* genotype closely related to *B. canis*, which previously had been detected in a raccoon from the Czech Republic^14^. Although canids are the expected main hosts, *B. canis* has been found in several other mammals, including cats, equids, and bats^45–49^. The second sequence from the present study (GenBank accession number: PP577797) was more closely related to *Babesia* spp. found in ruminants. This could suggest that either the *18S rRNA* is not a suitable marker for species differentiation or that some *Babesia* species are not as host-specific as formerly assumed. Both hypotheses are supported by a study screening wildcats from Romania, in which *B. canis* was detected. After analysis of the genetic markers *Cytochrome b* and *COI* the authors proposed a new species, but also confirmed the presence of *B. canis* in wildcats^45^.

Four to five phylogenetically distinct clades of *Babesia* spp. were described in raccoons in Canada, USA, and Japan^24,25^. Interestingly, none of these genotypes was detected in the present study. This wide range of genotypes could indicate that raccoons are suitable hosts for a variety of *Babesia* spp. or can carry the pathogen transiently and the species detected are mainly dependent on the geographic location and less specific for the raccoon as a host.

A serological survey in raccoons from Germany reported a prevalence of 15.7% for *A. phagocytophilum*, while another study based on PCR could not detect this pathogen in German raccoons at all^15,18^. Similarly, using PCR as the detection method, we also found a low frequency of infection (0.7%) with *A. phagocytophilum* in the present study, whereas in Poland (4.7%) and Czech Republic (11.4%) higher percentages of raccoons were reported to be affected^14,15^. Nevertheless, considering its notably higher prevalence in wild cervids from Germany (> 90%), the raccoon might be a negligible reservoir for this pathogen^42,43^.

The role of raccoons as a reservoir for *Mycoplasma* spp. and *Babesia* spp. must be investigated further, especially since the raccoon population is spreading and their synanthropic lifestyle is leading to closer contact with humans and domestic animals^50^. Furthermore, since the genotypes of the pathogens detected in raccoons are also found in other animal species, more research and monitoring are necessary to identify other possible reservoirs.

## Materials and methods

### Sample collection

Between 2019 and 2022, 285 wild raccoons were legally hunted for a study aiming to control the zoonotic nematode *Baylisascaris procyonis* in Germany^51,52^. We investigated carcasses from four German regions (Fig. 1): Eichsfeld, Thuringia (51° 20′ N/10° 10′ E; size of study area 800 km^2^; n = 65), Wittenberg, Saxony-Anhalt (51° 51′ N/12° 42′ E; 250 km^2^; n = 51), Upper Lusatia, Saxony (51° 16′ N/14° 44′ E; 800 km^2^; n = 144) and Swabian Jura, Baden-Württemberg (48° 47′ N/10° 02′ E; 450 km^2^; n = 23). Two individuals were of unknown origin. In total 151 males, 131 females, and 3 individuals of unknown sex were collected. Age of the animals was not determined or recorded. During dissection spleen samples were collected and stored in alcohol at − 20 °C until final processing at the University of Veterinary Medicine, Vienna.

### DNA extraction, PCR amplification, and sequencing

DNA was isolated from spleen samples of approximately 3 × 3 mm size using the QIAamp® 96 Virus QIAcube® HT Kit (QIAGEN, Hilden, Germany). Samples were incubated at 56 °C overnight and processed according to the manufacturer’s protocol. Samples were screened for the presence of various blood-associated pathogens using specific broad-range PCR assays (Table 1) targeting fragments of the mitochondrial *Cytochrome c oxidase subunit* I gene (*COI*) of Filarioidea; the *18S rRNA* gene of Piroplasmida and Trypanosomatida; the *16S rRNA* gene of *Mycoplasma* spp. and Anaplasmataceae; the 16S–23S rRNA intergenic spacer of *Bartonella* spp.; and the 23S–5S rRNA intergenic spacer of *Rickettsia* spp. To detect a broader range of pathogens, the following PCR assays targeted more than one pathogen genus: Filarioidea (*Onchocerca* spp., *Dirofilaria* spp., a.o.), Piroplasmida (*Babesia* spp., *Theileria* spp., a.o.), Trypanosomatida (*Trypanosoma* spp., *Leishmania* spp., a.o.), Anaplasmataceae (*Anaplasma* spp., *Ehrlichia* spp., a.o.). For PCR the GoTaq® DNA Polymerase (Promega, Walldorf, Germany) was used according to the manufacturer’s instructions. The exact time and temperature profiles as well as the concentrations can be found in the supplements. Positive and negative controls were used to validate results. PCR products were analysed by electrophoresis in 2% agarose gels stained with Midori Green Advance DNA stain (Nippon Genetics Europe, Germany). Positive samples were sent to LGC Genomics GmbH (Berlin, Germany) for purification and sequencing in both directions using the PCR primers. The forward and reverse sequences were aligned and electropherograms were visually inspected and edited using Bioedit v. 7.0.5.3^53^.

**Table 1:** Oligonucleotide sequences of primers used in the present study.

Target organism (genetic marker)	Primer sequences (5′ → 3′)	Product size	Annealing temperature	Reference
Filarioidea (*COI*)	COIint-F: TGA TTG GTG GTT TTG GTA A COIint-R: ATA AGT ACG AGT ATC AAT ATC	668 bp	45 °C	^54^
Piroplasmida (*18S rRNA*)	BTH-1F: CCT GAG AAA CGG CTA CCA CAT CT BTH-1R: TTG CGA CCA TAC TCC CCC CA	700 bp	68 °C	^55^
	GF2: GTC TTG TAA TTG GAA TGA TGG GR2: CCA AAG ACT TTG ATT TCT CTC	561 bp	60 °C	
Trypanosomatida (*18S rRNA*)	Tryp_18S_F: GTG GAC TGC CAT GGC GTT GA Tryp_18S_R: CAG CTT GGA TCT CGT CCG TTG A	1320 bp	56 °C	^56^
	Tryp_18S_F2: CGA TGA GGC AGC GAA AAG AAA TAG AG Tryp_18S_R2: GAC TGT AAC CTC AAA GCT TTC GCG	960 bp	56 °C	
*Mycoplasma* spp. (*16S rRNA*)	HBT-F: ATA CGG CCC ATA TTC CTA CG HBT-R: TGC TCC ACC ACT TGT TCA	600 bp	60 °C	^57^
Anaplasmataceae (*16S rRNA*)	EHR16SD_for: GGT ACC YAC AGA AGA AGT CC EHR16SR_rev: TAG CAC TCA TCG TTT ACA GC	345 bp	54 °C	^58^
*Bartonella* spp. (16S-23S rRNA)	bartgd_for: GAT GAT GAT CCC AAG CCT TC B1623_rev: AAC CAA CTG AGC TAC AAG CC	179 bp	60 °C	^59^
*Rickettsia* spp. (23S-5S rRNA)	ITS-F: GAT AGG TCG GGT GTG GAA G ITS-R: TCG GGA TGG GAT CGT GTG	350–550 bp	52 °C	^60^

### Phylogenetic analysis

The *Mycoplasma* and *Babesia* sequences obtained were subjected to BLAST searches on NCBI GenBank. For the phylogenetic analyses, nucleotide sequences available on the NCBI GenBank database were searched with the BLAST function, using one of the sequences obtained for each organism with the number of maximum target sequences set to 5000. For *Babesia* spp. the filter was set to 90–100% identity and 98–100% query coverage and for *Mycoplasma* spp. to 85–100% identity and 99–100% query coverage. The sequences were aligned and sorted using the default option (FFT–NS–2) in MAFFT v.7.520^61^. All sequences featuring obvious sequencing errors and ambiguous characters were removed from the alignment and were excluded from the analysis. To provide an overview of the diversity of haplotypes, Maximum Likelihood (ML) and Bayesian Inference (BI) trees were calculated for each organism based on alignments including 592 sequences (598 nucleotide positions) of *18S rRNA* for *Babesia* spp. and 553 sequences (608 nucleotide positions) of *16S rRNA* for *Mycoplasma* spp. Alignment gaps were removed using TrimAl v.1.3 (http://phylemon2.bioinfo.cipf.es/;^62^) and sequences were collapsed to haplotypes using DAMBE v.7.3.32^63^, leaving 167 haplotypes (436 nucleotide positions) for *Babesia* spp. and 162 haplotypes (538 nucleotide positions) for *Mycoplasma* spp. A sequence of *Hepatozoon canis* (GenBank accession number: KU893125) was used as an outgroup for the *Babesia* trees, and a sequence of *Mycoplasma gallisepticum* (GenBank accession number: MH539021) was used for the *Mycoplasma* trees. The ML bootstrap consensus trees (1000 replicates) were calculated using the W-IQ-TREE web server (http://iqtree.cibiv.univie.ac.at/;^64^) applying the models TVMe + I + G4 for *Babesia* spp. TIM2 + F + I + G4 for *Mycoplasma* spp., which were suggested as best fit for the data set in the model test according to the Bayesian inference criterion (BIC). The BI trees were calculated using MrBayes v.3.2.7^65^, applying the next complex model GTR + G + I, because the same models were not available in this program. The analyses were run for 10^6^ generations (Number of chains: 4), sampling every thousandth tree. The first 25% of trees were discarded as burn-in and a 50% majority-rule consensus tree was calculated based on the remaining 7500 trees. The ML and BI tree were joined, graphically prepared and provided with information on the countries and hosts in CorelDRAW 2024 (Corel, Ottawa, Canada).

## Supplementary Information

Supplementary Information.

## Data Availability

The datasets used and/or analysed during the current study are available from the corresponding author on reasonable request. All sequences were uploaded to NCBI GenBank (PP590044- PP590159 and PP577796- PP577798).
