# Supplementary material for: Detection and phylogenetic analysis of blood-associated pathogens from spleen samples of wild raccoons (Procyon lotor) in Germany
Source: Sci Rep. 2024 Dec 28;14:31232. doi: 10.1038/s41598-024-82581-7 (PMC11682041; doi:10.1038/s41598-024-82581-7)
Supplement: Supplementary file 1 — Supplementary Information. [file 41598_2024_82581_MOESM1_ESM.pdf]

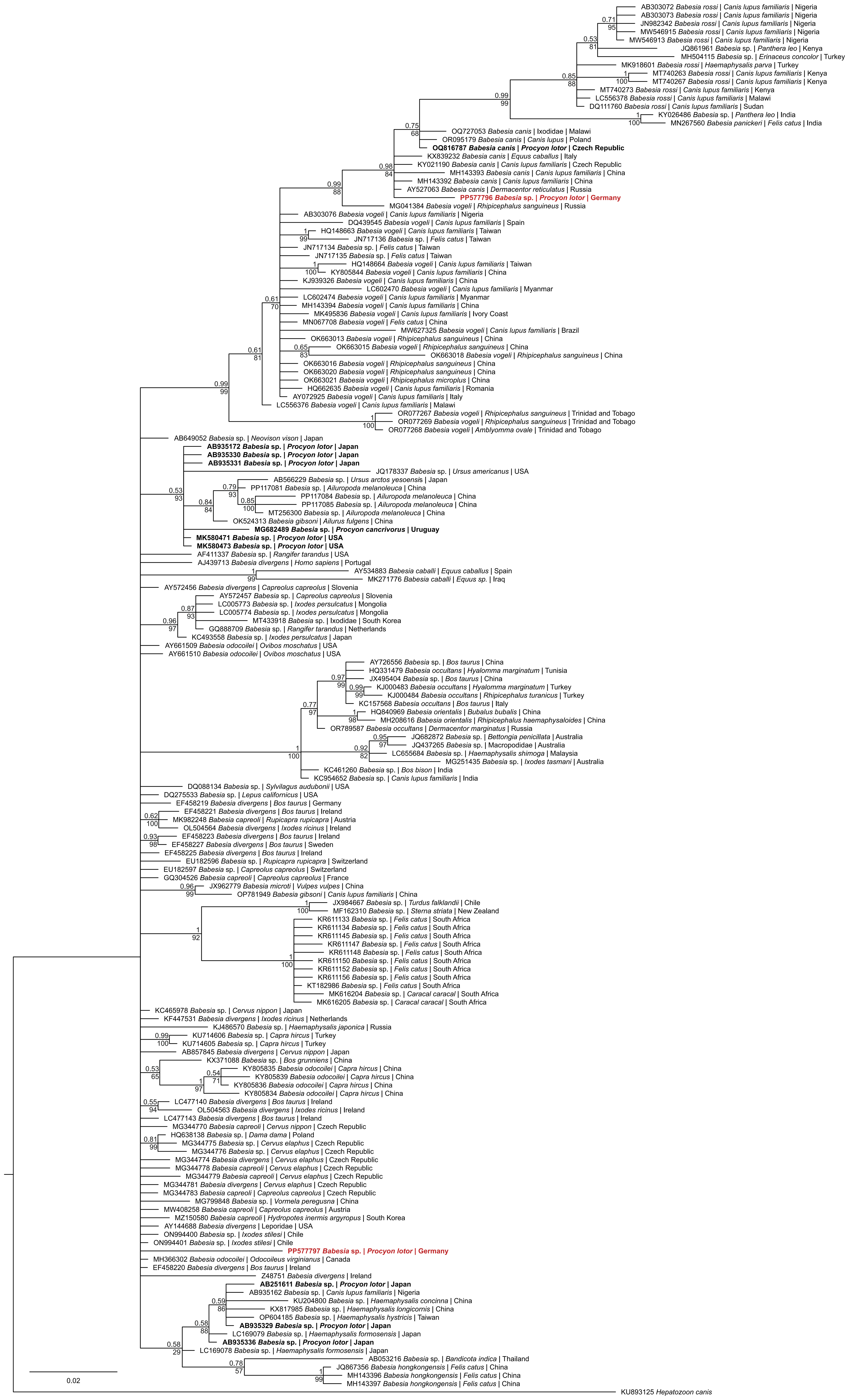

Supplement 1. Bayesian interference (BI) tree featuring *18S rRNA* (436 nucleotide positions) sequences of selected *Babesia* spp. Nodes are marked with BI posterior probabilities and maximum likelihood bootstrap values. Sequences which are written in bold are from *Procyon* spp. and sequences marked in red were obtained in this study. Scale bar indicates the expected mean number of substitutions per site according to the model of sequence evolution applied.

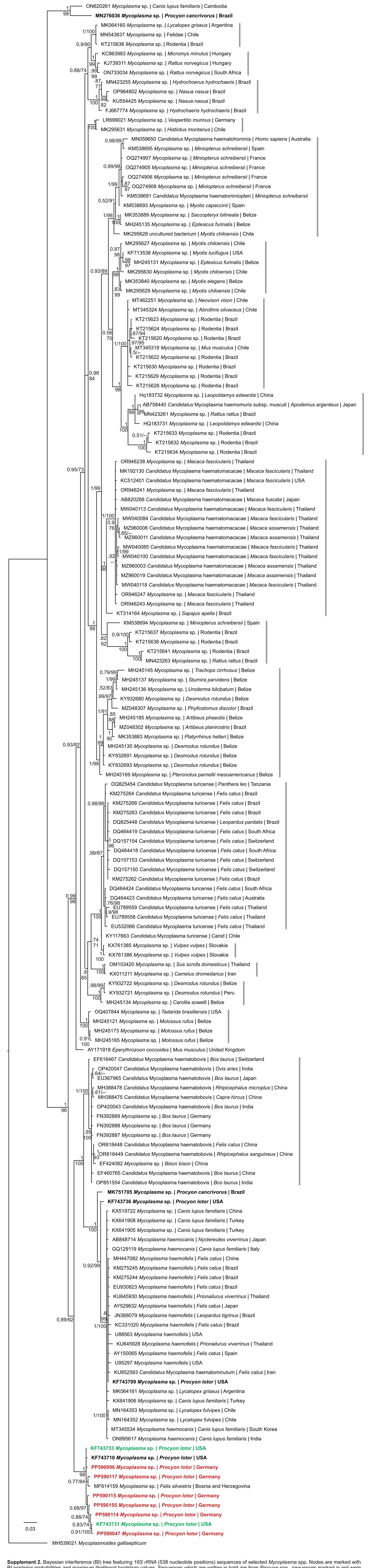

Supplement 2. Bayesian interference (BI) tree featuring *16S rRNA* (538 nucleotide positions) sequences of selected *Mycoplasma* spp. Nodes are marked with BI posterior probabilities and maximum likelihood bootstrap values. Sequences which are written in bold are from *Procyon* spp., sequences marked in red were obtained in this study, and sequences marked in green are identical to sequences obtained in this study. Grey bars indicate the clades that were collapsed in Fig. 2. Scale bar indicates the expected mean number of substitutions per site according to the model of sequence evolution applied.

Supplement 3. PCR protocols and cycling conditions used in the present study.

Target organism (genetic marker)	Mastermix protocol			Cycling Conditions		
	Reagents	Unit Reagent	Quantity (µl) per tube	Temperature (°C)	Time	Step
**Filarioidea (*COI*)**	H_2O		14.175	94	2’	Initial denaturation
	green reaction buffer	5 x	5	94	45’’	8 x
	dNTPs	25 mM	0.2	51	45’’	Decrease by 0.5°C
	TagPolymerase (GoTaq)	5 u/µl	0.125	72	1’30’’	
	Primer Forward	100 pmol/µl	0.25	94	45’’	25 x
	Primer Reverse	100 pmol/µl	0.25	45	45’’	
	Template	5 µl	5	72	1’30’’	
				72	7’	Final extension
**Piroplasmida (*18S rRNA*)**	H_2O		14.24			
**Nest 1**	green reaction buffer	5 x	5	94	2’	Initial denaturation
	dNTPs	25 mM	0.2	95	30’’	40 x
	TagPolymerase (GoTaq)	5 u/µl	0.125	68	1’	
	Primer Forward	100 pmol/µl	0.25	72	1’	
	Primer Reverse	100 pmol/µl	0.25	72	10’	Final extension
	Template	10 µl	5			
**Nest 2**	H_2O		13.75			
	green reaction buffer	5 x	5	94	2’	Initial denaturation
	dNTPs	25 mM	0.2	95	30’’	40 x
	TagPolymerase (GoTaq)	5 u/µl	0.2	60	1’	
	Primer Forward	100 pmol/µl	0.5	72	1’	
	Primer Reverse	100 pmol/µl	0.5	72	10’	Final extension
	Template	10 µl	5			

Supplement 3. PCR protocols and cycling conditions used in the present study (cont.).

Target organism (genetic marker)	Mastermix protocol			Cycling Conditions		
	Reagents	Unit Reagent	Quantity (µl) per tube	Temperature (°C)	Time	Step
**Trypanosomatida (*18S rRNA*)**	H_2O		14.675			
**Nest 1 + 2**	green reaction buffer	5 x	5	94	5'	Initial denaturation
	dNTPs	25 mM	0.2	94	1'	35 x
	TagPolymerase (GoTaq)	5 u/µl	0.125	56	1'	
	Primer Forward	10 pmol/µl	2	72	1'	
	Primer Reverse	10 pmol/µl	2	72	5'	Final extension
	Template	5 µl	1			
***Mycoplasma* spp. (*16S rRNA*)**	H_2O		12.675			
	green reaction buffer	5 x	5	94	2'	Initial denaturation
	dNTPs	25 mM	0.2	95	1'	40 x
	TagPolymerase (GoTaq)	5 u/µl	0.125	60	1'	
	Primer Forward	10 pmol/µl	1	72	1'	
	Primer Reverse	10 pmol/µl	1	72	7'	Final extension
	Template	5 µl	5			
**Anaplasmataceae (*16S rRNA*)**	H2O		11.175			
	green reaction buffer	5 x	5	95	2'	Initial denaturation
	dNTPs	25 mM	0.2	94	1'	30 x
	MgCl2	25 mM	1.5	54	3''	
	TagPolymerase (GoTaq)	5 u/µl	0.125	72	30''	
	Primer Forward	10 pmol/µl	1	72	5'	Final extension
	Primer Reverse	10 pmol/µl	1			
	Template	5 µl	5			

Supplement 3. PCR protocols and cycling conditions used in the present study (cont.).

Target organism (genetic marker)	Mastermix protocol			Cycling Conditions		
	Reagents	Unit Reagent	Quantity (µl) per tube	Temperature (°C)	Time	Step
Bartonella **spp. (16S-23S rRNA)**	H_2O		12.675			
	green reaction buffer	5 x	5	95	10’	Initial denaturation
	dNTPs	25 mM	0.2	95	15’’	30 x
	TagPolymerase (GoTaq)	5 u/µl	0.125	60	1’	
	Primer Forward	20 pmol/µl	1	72	20’’	
	Primer Reverse	20 pmol/µl	1	72	5’	Final extension
	Template	5 µl	5			
Rickettsia **spp. (23S-5S rRNA)**	H_2O		12.675			
	green reaction buffer	5 x	5	96	4’	Initial denaturation
	dNTPs	25 mM	0.2	94	1’	35 x
	TagPolymerase (GoTaq)	5 u/µl	0.125	52	1’	
	Primer Forward	10 pmol/µl	1	72	2’	
	Primer Reverse	10 pmol/µl	1	72	3’	Final extension
	Template	5 µl	5			
